# The Probiotic *Lactobacillus reuteri* Preferentially Synthesizes Kynurenic Acid from Kynurenine

**DOI:** 10.3390/ijms25073679

**Published:** 2024-03-26

**Authors:** Robert Schwarcz, Ann Foo, Korrapati V. Sathyasaikumar, Francesca M. Notarangelo

**Affiliations:** Maryland Psychiatric Research Center, Department of Psychiatry, University of Maryland School of Medicine, Baltimore, MD 21228, USA; afoo@umces.edu (A.F.); saikumar@som.umaryland.edu (K.V.S.);

**Keywords:** cognition, gut–brain axis, kynurenine pathway, kynurenine aminotransferases, psychiatric disorders

## Abstract

The gut–brain axis is increasingly understood to play a role in neuropsychiatric disorders. The probiotic bacterium *Lactobacillus (L.) reuteri* and products of tryptophan degradation, specifically the neuroactive kynurenine pathway (KP) metabolite kynurenic acid (KYNA), have received special attention in this context. We, therefore, assessed relevant features of KP metabolism, namely, the cellular uptake of the pivotal metabolite kynurenine and its conversion to its primary products KYNA, 3-hydroxykynurenine and anthranilic acid in *L. reuteri* by incubating the bacteria in Hank’s Balanced Salt solution in vitro. Kynurenine readily entered the bacterial cells and was preferentially converted to KYNA, which was promptly released into the extracellular milieu. De novo production of KYNA increased linearly with increasing concentrations of kynurenine (up to 1 mM) and bacteria (10^7^ to 10^9^ CFU/mL) and with incubation time (1–3 h). KYNA neosynthesis was blocked by two selective inhibitors of mammalian kynurenine aminotransferase II (PF-048559989 and BFF-122). In contrast to mammals, however, kynurenine uptake was *not* influenced by other substrates of the mammalian large neutral amino acid transporter, and KYNA production was not affected by the presumed competitive enzyme substrates (glutamine and α-aminoadipate). Taken together, these results reveal substantive qualitative differences between bacterial and mammalian KP metabolism.

## 1. Introduction

The gut–brain axis has recently emerged as a major topic of interest in neurobiology, especially with regard to the pathophysiology of neuropsychiatric disorders. Mounting evidence supports the hypothesis that gut microbiota regulate brain development and behavior [1,2,3,4,5] and that dysbiosis contributes to cognitive deficits and other behavioral abnormalities [6,7,8] and may be critically involved in a variety of psychiatric diseases [9,10,11,12].

Studies in germ-free and antibiotic-treated rodents suggest that the essential amino acid tryptophan plays a special role in this context [13]. In the mammalian gut, most dietary tryptophan is either absorbed in the small intestine or locally converted to biologically active metabolites that then enter the circulation [14]. About 5% of tryptophan is metabolized by specific gut bacteria, mostly to indole derivatives [15,16,17], and, notably, elevated circulating tryptophan levels are associated with a decrease in gut microbiota [13,18].

**Figure 1:**
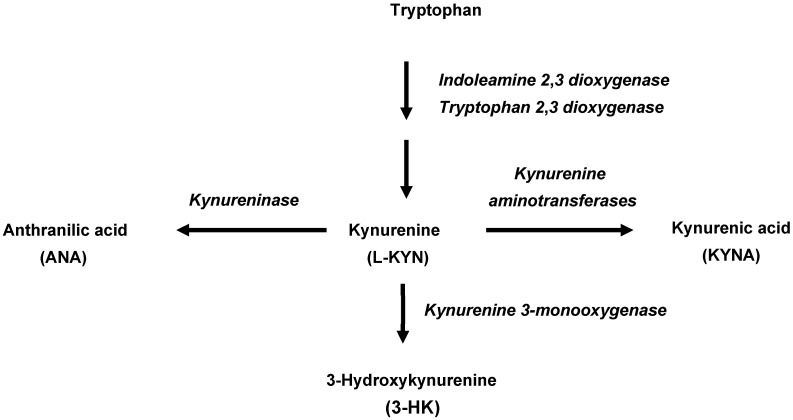
Initial enzymatic processes involved in the kynurenine pathway of tryptophan metabolism in mammals.

In mammals, 95% of dietary tryptophan is degraded via the kynurenine pathway (KP), which is named after the pivotal metabolite L-kynurenine (L-KYN). Conversion of tryptophan to L-KYN is catalyzed by indoleamine 2,3-dioxygenases (IDO 1 and IDO 2), which are present in the intestine, brain and immune cells and activated by inflammatory stimuli and tryptophan 2,3-dioxygenase (TDO), which is mostly expressed in the liver. Subsequently, L-KYN is metabolized to the free radical generator 3-hydroxykynurenine (3-HK) by kynurenine 3-monooxygenase or to anthranilic acid (ANA) by kynureninase (Figure 1). Of special significance for brain function and dysfunction [19,20,21,22,23], L-KYN is also converted by kynurenine aminotransferases (KATs) to kynurenic acid (KYNA), an antagonist of N-methyl-D-aspartate (NMDA; [24,25], and α7 nicotinic acetylcholine (α7nACh; [26] receptors and an agonist at an orphan G-protein-coupled receptor (GPR35; [27]. Interestingly, KYNA can also regulate the immune system through its agonistic effects on the aryl hydrocarbon receptor (AhR; [28] and is an effective scavenger of reactive oxygen species [29,30].

Although the formation of KP metabolites by intestinal microbiota impacts host function [31], and in spite of the fact that experimental manipulation of the gut microbiota affects KP metabolism in the host [6,32,33,34,35,36], only relatively little is known about the ability of individual intestinal bacteria to express functional KP enzymes and to produce KYNA, 3-HK and ANA [37,38,39,40].

*Lactobacillus (L.) reuteri*, a well-studied probiotic bacterium that colonizes the gastrointestinal tract of both humans and animals, can influence the composition of the gut microbiota [41] and has been shown to exert a remarkable range of beneficial effects in both humans and relevant animal models. Notably, *L. reuteri* administration affects neurodevelopment and prevents social deficits and depressive-like behaviors in experimental animals [41,42,43,44,45,46,47]. Interestingly, though the underlying mechanisms have not been identified so far, *L. reuteri* is able to normalize the impaired plasma levels of KP metabolites caused by chronic stress and to improve associated behavioral abnormalities [35].

The present study was designed to examine the intricacies of KP metabolism in this translationally relevant bacterial species in greater depth. To this end, we investigated the ability of *L. reuteri* to accumulate exogenous L-KYN and to produce KYNA, 3-HK and ANA under a variety of experimental conditions in vitro.

## 2. Results

### 2.1. L-KYN Uptake into Live L. reuteri

**Figure 2:**
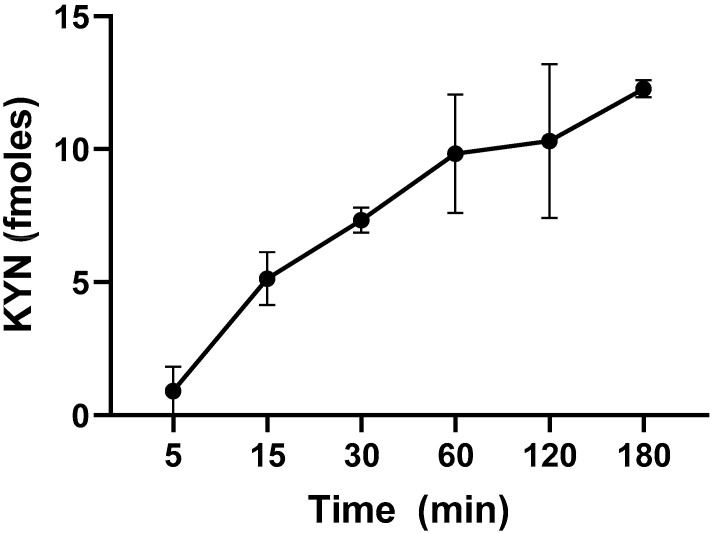
Time-dependence of L-KYN uptake into live *L. reuteri* (10^9^ CFU/mL). Bacteria were incubated in the presence of 100 µM L-KYN and 0.2 µCi ^3^H-L-KYN (see text for experimental details). Data are the mean ± SEM of 3 experiments.

To characterize KP metabolism in live *L. reuteri*, we first tested the ability of L-KYN to enter the cells. Incubation resulted in increased levels of intracellular L-KYN, starting at 5 min and slowly rising until 180 min, indicating that L-KYN rapidly enters and accumulates in the bacterial cells (Figure 2). Under standard conditions (1 h incubation at 37 °C), significantly less L-KYN was recovered from heat-inactivated bacteria (9.8 ± 1.3 vs. 3.1 ± 1.0 fmoles, *n* = 3; *p* < 0.05; Student’s *t*-test). 

We next tested selected amino acids that are known to compete with L-KYN as substrates of the large neutral amino acid transporter (LAT) in mammals [48,49] for their ability to affect L-KYN uptake into bacterial cells (Table 1). Unexpectedly, none of the tested compounds affected L-KYN uptake at a concentration of 5 mM (*p* > 0.05; one-way ANOVA, followed by Bonferroni’s post hoc test). Five mM BCH, an inhibitor of L-type amino acid transporters including the LAT [50], also failed to affect L-KYN uptake (*p* > 0.05; one-way ANOVA, followed by Bonferroni’s post hoc test), indicating substantive qualitative differences between L-KYN uptake into bacterial and mammalian cells.

### 2.2. Basal Levels and De Novo Production of KYNA, 3-HK and ANA 

Next, we examined the presence of endogenous levels of KP metabolites in live *L. reuteri* and their de novo production after incubation with L-KYN for 3 h. Under these conditions, the basal levels of KYNA (2.4 ± 1.7 fmoles/µL, *n* = 4) and ANA (1.9 ± 0.7 fmoles/µL, *n* = 3) were clearly measurable, but basal 3-HK levels were below our limit of detection (<0.5 fmoles/µL).

**Figure 3:**
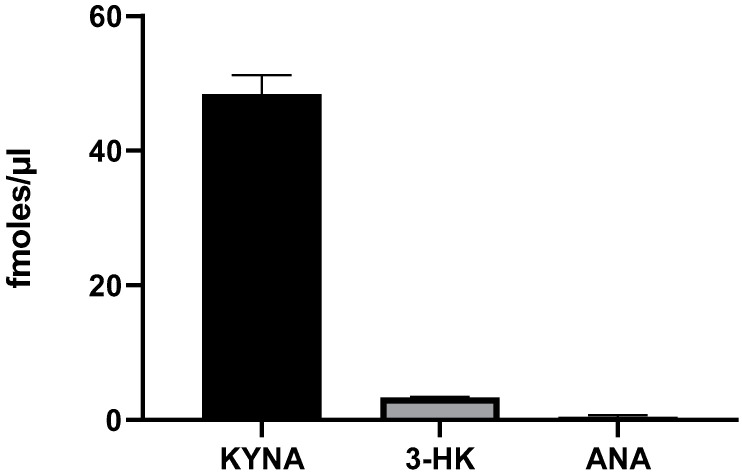
De novo production of KYNA, 3-HK and ANA by live *L. reuteri* (10^8^ CFU/mL). Bacteria were incubated for 3 h with 100 µM L-KYN, and metabolites were recovered from the extracellular milieu. See text for experimental details. Data are the mean ± SEM of 3–4 experiments.

Incubation with 100 µM L-KYN induced a significant increase in the levels of KYNA (*p* < 0.0001; Student’s *t*-test) and raised the levels of 3-HK above the detection limit (2.8 ± 0.6 fmoles/µL); however, it did not significantly elevate the levels of ANA (*p* > 0.05; Student’s *t*-test), demonstrating that L-KYN in *L. reuteri* is preferentially converted to KYNA (Figure 3).

In view of these results, subsequent experiments focused exclusively on evaluating the de novo synthesis of KYNA and its regulation.

### 2.3. Optimization of De Novo synthesis of KYNA from L-KYN by Live L. reuteri

**Figure 4:**
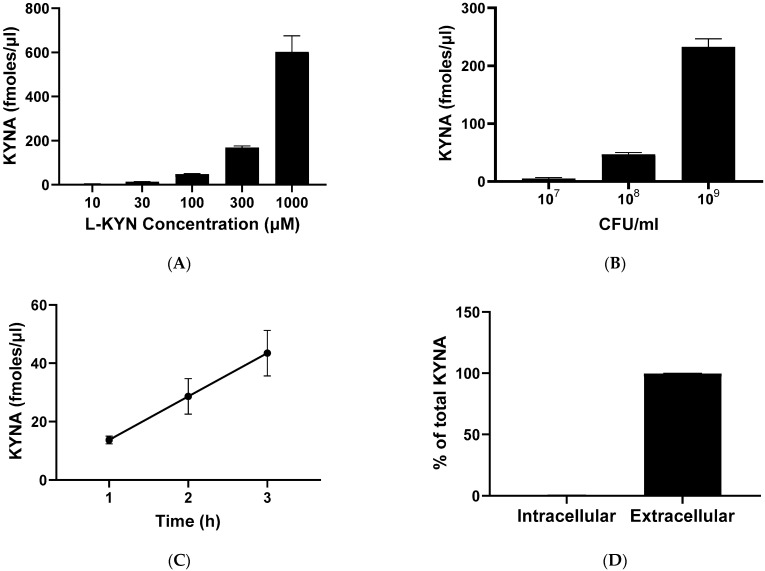
De novo synthesis of KYNA from L-KYN in live *L. reuteri*. (**A**–**C**): concentration of KYNA recovered from the extracellular milieu (see text for experimental details). (**A**) KYNA production in bacteria (10^8^ CFU/mL) incubated for 3 h with increasing concentrations of L-KYN. (**B**) KYNA formation after incubation of different concentrations of *L. reuteri* with 100 µM L-KYN for 3 h. (**C**) Effect of incubation time on KYNA neosynthesis using 100 µM L-KYN and 10^8^ CFU/mL *L. reuteri*. (**D**) Percentage of KYNA detected in the intracellular vs. the extracellular compartment following a 3 h incubation of *L. reuteri* (10^8^ CFU/mL) with 100 µM L-KYN. In all cases, data are the mean ± SEM of 3–4 experiments. See text for experimental details.

To determine the optimal conditions for the neosynthesis of KYNA production in live *L. reuteri*, we tested different L-KYN and bacterial concentrations as well as various incubation times (Figure 4). A dose-dependent elevation in KYNA levels was observed with increasing L-KYN concentrations (ranging from 10 to 1000 µM; Figure 4A) and bacterial density (from to 10^7^ to 10^9^ CFU/mL; Figure 4B). Increasing the incubation time from 1 to 3 h linearly raised KYNA formation *(*Figure 4C), and 99.6% of total KYNA produced from L-KYN was released into the extracellular milieu (Figure 4D). Longer incubation times (tested up to 24 h) failed to result in further increases. Based on these results, all subsequent experiments were conducted using 10^8^ CFU/mL bacteria, which were incubated for 3 h with 100 µM L-KYN.

In light of the ability of bacteria—in contrast to mammalian cells—to synthesize D-amino acids [51,52], we also investigated the production of KYNA from D-KYN. Incubation of *L. reuteri* with 100 µM D-KYN under standard experimental conditions (10^8^ CFU/mL bacteria, 3 h) caused a significant elevation in KYNA over endogenous levels (*p* < 0.01; Student’s *t*-test); however, the increase in extracellular KYNA was only ~30% of the effect resulting from incubation with L-KYN (18.1 ± 3.5 vs. 65.1 ± 3.5 fmoles/µL, *n* = 3; *p* < 0.05; Student’s *t*-test).

Notably, no de novo formation of KYNA was detected when *L. reuteri* was incubated with 1 mM of L-tryptophan under standard conditions.

### 2.4. Pharmacological Regulation of KYNA Production in Live L. reuteri 

**Figure 5:**
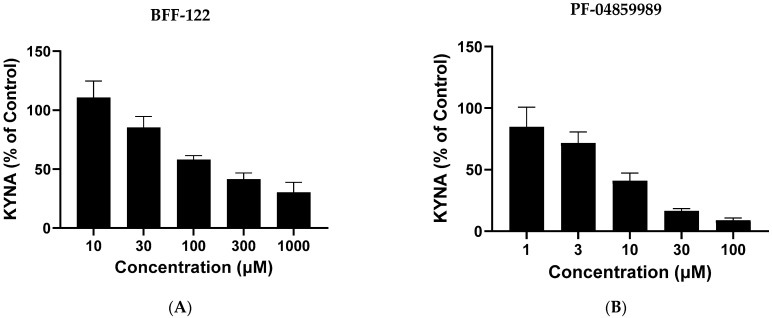
Dose-dependent inhibition of KYNA neosynthesis by BFF-122 (**A**) and PF-04859989 (**B**). *L. reuteri* (10^8^ CFU/mL) was incubated for 3 h with 100 µM L-kynurenine. Control values: 48.4 ± 2.8 fmoles/µL. See text for experimental details. Data are the mean ± SEM of 3 experiments.

To examine the role of KAT enzymes (KAT I, KAT II, KAT III and KAT IV; [53,54] in KYNA formation in live *L. reuteri*, we tested the effect of endogenous amino acids, which are known to compete with L-KYN as respective substrates of the mammalian enzymes (glutamine for KAT I and KAT III, α-aminoadipate for KAT II and aspartate for KAT IV), as well as the non-specific aminotransferase inhibitor AOAA and the synthetic KAT II inhibitors BFF-122 [55] and PF-04859989 [56] (Table 2). Whereas 1 mM AOAA essentially totally prevented the de novo production of KYNA from L-KYN (99.7%, *p* < 0.0001) and 10 mM aspartate induced a small reduction (~25%, *p* < 0.01), neither glutamine nor α-aminoadipate (both at 10 mM) had an effect (*p* > 0.05). Interestingly, both BFF-122 (1 mM) and PF-04859989 (100 µM) significantly inhibited the bacterial neosynthesis of KYNA (*p* < 0.0001). Subsequent dose-dependency experiments showed higher efficacy of PF-04859989 compared to BFF-122 (>90% and <50% inhibition, respectively, at 100 µM; Figure 5).

We also studied the pro-cognitive and antioxidant compound N-acetylcysteine (NAC), which inhibits mammalian KAT II both in vitro and in vivo [57]. NAC showed only weak activity, with less than 40% inhibition of KYNA neosynthesis at 10 mM (*n* = 6; *p* < 0.05; Student’s *t*-test). To examine, more generally, the possible involvement of oxidative processes in the de novo formation of KYNA from L-KYN in live *L. reuteri*, we then tested the antioxidant ascorbic acid (500 µM) under the same experimental conditions. Ascorbic acid had no significant effect on KYNA production (*n* = 3; *p* > 0.05; Student’s *t*-test).

Finally, we examined the ability of several 2-oxoacids, which are established co-substrates of KATs and readily stimulate KYNA synthesis in mammalian tissues [58]. When added to the incubation buffer at a final concentration of 5 mM, only some of the compounds significantly enhanced L-KYN conversion to KYNA in the live bacteria, with α-ketoisovalerate having the greatest effect of the 2-oxoacids tested (increase to ~190% of control values; *p* < 0.0001). In contrast, the inclusion of oxaloacetate in the incubation medium unexpectedly caused a *reduction* in KYNA neosynthesis (*p* < 0.05) (Table 3).

### 2.5. KYNA Production in Homogenized Bacteria

To examine the de novo formation of KYNA from L-KYN in lysed cells, sonicated bacterial tissue was incubated under conditions that are optimal for the mammalian KAT II enzyme [59]. L-KYN was readily converted to KYNA and, as in live bacteria, 10 mM glutamine caused only a small, non-significant reduction in KYNA neosynthesis from L-KYN in the homogenate (cf. Table 2 and Table 4). However, the addition of α-aminoadipate or aspartate (both at 10 mM) had stronger effects in the homogenized bacteria, with reductions in KYNA formation of ~20% and ~70%, respectively (*p* < 0.05 and *p* < 0.0001). Notably, in contrast to their quantitatively different effects in live bacteria, the two synthetic KAT II inhibitors BFF-122 and PF-04859989 (both at 1 mM) had similar potency in bacterial homogenates (~95% and ~88% inhibition, respectively) (both *p* < 0.0001) (cf. Table 2 and Table 4).

## 3. Discussion

The present study was designed to evaluate the role of the probiotic bacterium *L. reuteri* in regulating KP metabolism and, in particular, its ability to synthesize KYNA, 3-HK and ANA from KYN. We demonstrated that L-KYN rapidly entered the bacterial cells, but its uptake was not affected by other substrates of mammalian LAT. Although we were able to detect endogenous levels of both KYNA and ANA in live bacteria, our findings revealed that L-KYN is preferentially metabolized to KYNA, which is then promptly released into the extracellular milieu.

Interestingly, we did not observe de novo formation of KYNA from tryptophan under our experimental conditions. Although not examined in the present study, *L. reuteri*, like several other intestinal bacteria, may, therefore, preferentially convert tryptophan to a number of biologically active indoles instead of forming KP metabolites [14]. On the other hand, *L. reuteri* could generate KYNA from D-KYN, though with lower efficiency than from L-enantiomer. This finding clearly deserves further scrutiny and may be of (patho)physiological relevance in view of the fact that bacteria, unlike eukaryotes, have the ability to synthesize D-amino acids [51,60]. Also of interest in this context, D-KYN is a better precursor of KYNA in *L. reuteri* than in rodents or humans [61,62]. 

In mammals, L-KYN enters cells through LATs, which are able to transport both branched (valine, leucine, isoleucine) and aromatic (tryptophan, phenylalanine) amino acids, all of which compete for entrance into the cells [48,49]. Although L-KYN was shown here to rapidly enter the bacterial cells, its uptake was not inhibited by tryptophan or other presumably competing amino acids. Similarly, the LAT inhibitor BCH, which effectively interferes with L-KYN uptake in both rat brain slices in vitro and mouse brain in vivo [50], did not affect L-KYN uptake into *L. reuteri*, indicating that the bacterial transporter differs substantively from the mammalian LAT. 

Although not studied systematically so far, attempts to examine enzymatic L-KYN degradation in individual bacteria have revealed remarkable qualitative strain differences. For example, both *Cytophaga hutchinsonii* and *Pseudomonas fluorescens* express kynurenine 3-monooxygenase, the enzyme responsible for the synthesis of 3-HK [63,64], *Pseudomonas fluorescens* also contains kynureninase to produce ANA [65,66], and *Pseudomonas aeruginosa* is capable of enzymatically producing all three primary L-KYN metabolites, i.e., KYNA, 3-HK and ANA [37] (cf. Figure 1). In the present study, we observed that *L. reuteri* preferentially converts L-KYN to KYNA and appears to contain very little kynureninase and kynurenine 3-monooxygenase. These results also suggest that the low endogenous levels of 3-HK and ANA in *L. reuteri* may be related to non-enzymatic degradative processes [67] and, in the case of ANA, may involve alternative synthetic routes [68]. 

Although the presence of KAT in bacteria has been known for decades [69], the examination of bacterial KYNA neosynthesis has attracted only limited interest so far [37,38,70]. The dominant conversion of L-KYN to KYNA seen in *L. reuteri* prompted us to study this mechanism in greater detail. The fact that ascorbic acid did not significantly reduce the bacterial formation of KYNA suggested that KYNA synthesis from L-KYN in *L. reuteri* was not caused by the non-enzymatic oxidation of L-KYN [71,72] but was mainly enzymatic in nature. Although confirmed in principle by the very effective blockade of KYNA formation by AOAA, the mechanism of bacterial KYNA production showed major qualitative differences from mammalian cells, however [59]. Thus, glutamine, a highly competitive substrate of mammalian KAT I (= glutamine aminotransferase), was unable to inhibit the de novo KYNA production in both live and homogenized bacteria, even at a high concentration (10 mM). Similarly, 10 mM α-aminoadipate, a competing substrate of mammalian KAT II (=α-aminoadipate aminotransferase), did not interfere with KYNA formation from L-KYN in live *L. reuteri* and caused only a small decrease (~20%) in homogenized bacteria. Interestingly, though, two synthetic inhibitors of mammalian KAT II (BFF-122 and PF-04859989; [55,56] substantially reduced KYNA production. These results, as well as the modest but significant effects caused by the addition of aspartate, a relatively weak inhibitor of mammalian KAT II [73], suggest that bacterial enzyme(s) bearing some similarity to mammalian KAT II or KAT IV [53] account for the neosynthesis of KYNA from L-KYN in *L. reuteri*. The distinct nature of this enzymatic process, which is further supported by the fact that several 2-oxoacids known to serve as amino-acceptors of mammalian KATs [74] failed to show the expected effects in the present study, clearly requires further clarification. Notably, future experiments should consider the growth rate and gene activity of *L. reuteri*, which were not taken into account in the present study.

The present results raise the possibility that KYNA plays a role in the remarkable beneficial effects of *L. reuteri* administration in both humans and rodents. Thus, through its antioxidant properties and/or by targeting several receptors with critical roles in both physiology as well as pathology, an elevation in KYNA levels may have considerable functional consequences in both the periphery and brain (cf. Introduction). For example, increased formation and release of KYNA by *L. reuteri* may regulate the enteric nervous system [75], affect the growth and viability of probiotic bacteria in the digestive system [76], alleviate various gastrointestinal pathologies [77] and have anti-inflammatory effects by inhibiting Th17 cell differentiation and the increase in TNFα in monocytes and leukocytes [20,78]. Notably, though the mechanistic link clearly needs to be investigated further, KYNA generated by *L. reuteri*—alone or in combination with other probiotics—may also participate in the attenuation of depressive-like symptoms associated with chronic stress [41] and in the reduction of obesity-related behavioral impairments and related microglial activation [42,43,46]. 

## 4. Materials and Methods

### 4.1. Materials

*L. reuteri* (F 275^T^ = ATCC 23272^T^ = DSM 20016^T^ = JCM 1112^T^ = LMG 9213^T^ = LMG 13557^T^) bacteria were obtained from the American Type Culture Collection (Manassas, VA, USA). ^3^H-L-kynurenine (^3^H-KYN) (16 Ci/mmol) was purchased from Amersham (Buckinghamshire, UK). Aminooxyacetic acid (AOAA), aspartate, α-aminoadipate, glutamine, pyruvate, α-ketoglutarate, α-ketoisocaproate, α-ketoisovalerate, oxaloacetate, 2-amino-2-norbornanecarboxylic acid (BCH) and PF-04859989 were obtained from Sigma-Aldrich (St. Louis, MO, USA). L-Kynurenine sulfate (L-KYN) was acquired from Sai Advantium (Hyderabad, India) and D-kynurenine sulfate (D-KYN) from Shanghai Hanhong Chemical Co. (Shanghai, China). BFF-122 [(S)-(-)-9-(4-aminopiperazine-1-yl)-8-fluoro-3-methyl-6-oxo-2,3,5,6-tetrahydro-4H-1-oxa-3a-azaphenalene-5-carboxylic acid] was kindly provided by Dr. Y. Kajii (Mitsubishi-Tanabe Pharma Corp., Yokohama, Japan). All other chemicals used were purchased from commercial suppliers and were of the highest available purity.

### 4.2. Preparation of the Bacteria

*L. reuteri* bacteria were grown on deMan, Rogosa and Sharpe (MRS) agar (Hardy Diagnostics, Santa Maria, CA, USA) for 24 h at 37 °C. The bacteria were then transferred to an MRS broth (Hardy Diagnostics) and quantified by serial dilution plating and counting colonies. Bacteria were diluted to 10^10^ or 10^9^ CFU/mL for storage, aliquoted and frozen at −80 °C. For all assays, bacterial aliquots were thawed at room temperature, centrifuged (6000× *g*, 2 min), rinsed with cold sterile Hank’s Balanced Salt Solution (HBSS; containing 1.0 g/L glucose, 0.011 g/L phenol red and 0.35 g/L sodium bicarbonate, pH 7.1–7.5; H9269; Sigma-Aldrich) and then resuspended in HBSS and diluted to the concentrations used in the respective assays. 

The viability of the bacteria under standard experimental conditions (10^8^ CFU/mL live bacteria in HBSS, 100 µM KYN, 3 h incubation) was evaluated by serial dilution plating and counting colonies. 

### 4.3. L-KYN Uptake by Live Bacteria

^3^H-KYN was purified by high-performance liquid chromatography (HPLC) prior to its use in uptake experiments [79]. L-KYN uptake was assessed by incubating 10^9^ CFU/mL live *L. reuteri* in HBSS at 37 °C for 5 to 180 min in the presence of 0.2 µCi of ^3^H-KYN (20 µL), 100 µM non-radioactive L-KYN (10 µL) and water (replaced by dissolved test compounds when indicated), in a total volume of 200 µL. Blank values were routinely obtained by incubating the bacteria on ice. Inactivated bacteria (cells heated for 2 min at 100 °C before incubation) were examined in some experiments. Incubations were performed using a Roto-Therm H2020 Fixed Speed Incubated Tube Rotator (Benchmark Scientific, Edison, NJ, USA) at 24 rpm. 

Following incubation, the assay mixture was immediately centrifuged (6000× *g*, 2 min), and the supernatant was discarded. The remaining pellet was placed on ice, and residual radioactivity was eliminated by adding 500 µL of cold HBSS, gentle mixing and further centrifugation (6000× *g*, 2 min). After removal of the supernatant, the pellet was suspended in 200 µL HBSS and transferred to vials containing 10 mL scintillation fluid. Radioactivity was measured by liquid scintillation spectrometry (Packard Tri-Carb 2200CA LCA, Perkin Elmer, Boston, MA, USA).

### 4.4. KYNA, 3-HK and ANA Production by Live Bacteria 

De novo synthesis of KYNA, 3-HK and ANA from L-KYN by live bacteria was assessed by incubation at 37 °C in a total volume of 200 µL [160 µL of bacteria in HBSS, 20 µL of L-KYN and 20 µL of water (replaced by KAT inhibitors or 2-oxoacids in some experiments)] using a Roto-Therm H2020 Fixed Speed Incubated Tube Rotator at 24 rpm. Incubation times (1, 2 or 3 h), bacterial density (10^7^–10^9^ CFU/mL) and L-KYN concentrations (10–1000 µM) varied during method development. The reaction was terminated by the addition of 40 µL of 50% trichloroacetic acid (TCA). Samples were then centrifuged (15,800× *g*, 10 min), and the supernatant was removed and analyzed as detailed below. Blanks were obtained by incubating bacteria in the absence of L-KYN. 

To measure intracellular KYNA, the pellet containing the bacterial cells was resuspended in 200 µL of water and sonicated. After centrifugation (15,800× *g*, 5 min), KYNA was determined in the supernatant. 

### 4.5. KYNA Synthesis in Lysed L. reuteri Cells

The neosynthesis of KYNA was also examined in homogenized *L. reuteri* cells. To this end, frozen bacteria were thawed and centrifuged (6000× *g*, 10 min). The MRS broth was replaced with 0.5 M Tris-acetate buffer (pH 8.0) to obtain a final bacterial concentration of 10^8^ CFU/mL, and the solution was sonicated to lyse the bacterial cells. Assays were performed in a total volume of 200 µL [80 µL of sonicated 10^8^ CFU/mL bacteria, 100 µL of assay cocktail (100 µM L-KYN, 80 µM pyridoxal-5′-phosphate and 1 mM pyruvate in 150 mM Tris-acetate buffer, pH 7.4) and 20 µL of water (replaced by KAT inhibitors in some experiments)]. Blanks were obtained by adding the non-specific aminotransferase inhibitor AOAA (final concentration: 1 mM) to the solution. After incubation for 3 h at 37 °C, the reaction was terminated by the addition of 20 µL of cold 50% trichloroacetic acid (TCA) and 1 mL 0.1 N HCl. Samples were kept on ice and centrifuged (15,800× *g*, 10 min), and KYNA was analyzed in the supernatant.

### 4.6. KYNA, 3-HK and ANA Measurement 

KYNA quantification was performed by high-performance liquid chromatography (HPLC) with fluorescent detection. In total, 20 µL of the supernatant was injected onto a BDS Hypersil C18 column (100 mm × 4.6 mm, particle size 3 µm; Thermo Fisher Scientific, Waltham, MA, USA) with a mobile phase consisting of 3% acetonitrile, 250 mM sodium acetate and 50 mM zinc acetate (pH 6.2) at a flow rate of 1 mL/min. Fluorescence detection was performed using a Perkin Elmer Series 200a instrument (Perkin Elmer, Shelton, CT, USA) (excitation: 344 nm, emission: 398 nm). The retention time was approximately 8 min.

3-HK quantification was performed by HPLC with electrochemical detection. To this end, 20 µL of the supernatant was injected onto a C18 reverse phase column (HR-80; 80 mm × 4.6 mm; particle size 3 µm; Thermo Fisher Scientific) with a mobile phase consisting of 1.5% acetonitrile, 0.9% trimethyl amine, 0.59% phosphoric acid, 0.27 mM EDTA and 8.9 mM sodium heptane sulfonic acid at a flow rate of 0.5 mL/min. 3-HK was detected electrochemically using an HTEC 500 detector (Eicom, San Diego, CA, USA) and had a retention time of approximately 11 min.

For ANA detection, 20 μL of the supernatant was applied to a 5 μm C_18_ reverse phase column (Adsorbosil; 150 mm × 4.6 mm; Dr. Maisch GmbH, Ammerbuch, Germany) using a mobile phase containing 100 mM sodium acetate (pH 5.8) and 1% acetonitrile at a flow rate of 1.0 mL/min. ANA was detected fluorimetrically in the eluate (excitation: 340 nm; emission: 410 nm; 2475 fluorescence detector; Waters, Milford, MA, USA). The retention time was approximately 6 min [80].

### 4.7. Statistical Analysis

All data are expressed as the mean ± SEM. Statistical analyses were performed using Graphpad Prism 9 (San Diego, CA, USA). Student’s *t*-test or one-way ANOVA followed by Bonferroni’s post hoc test was used to determine significance in selected experiments. A *p* value < 0.05 was considered significant.

## 5. Conclusions

In summary, the present in vitro study not only provided further evidence of the well-established ability of gut microbiota to metabolize the major tryptophan metabolite L-KYN but revealed that *L. reuteri*, in contrast to other bacteria, preferentially synthesizes KYNA, which is then promptly released into the extracellular milieu. Notably, an assessment of the underlying biochemical mechanisms revealed substantive qualitative differences from mammalian KP metabolism. As the administration of *L. reuteri* has remarkably beneficial effects in both animals and humans and since KYNA is increasingly understood to play substantive roles in mammalian biology, our results may have significant translational implications. Future studies should, therefore, be designed to manipulate KYNA production in *L. reuteri* and selected other bacteria by pharmacological and genetic means and to examine the physiological and pathological consequences of such bacterial modifications in the host in vivo.

## Figures and Tables

**Table 1:** Effects of selected compounds (final concentration: 5 mM) on L-KYN uptake into live *L. reuteri*.

Test Compound	Kynurenine Uptake (% of Control)
Leucine	115.3 ± 13.5
Tryptophan	109.7 ± 14.0
Valine	111.5 ± 11.9
Isoleucine	123.1 ± 4.4
Phenylalanine	131.5 ± 11.1
2-Amino-2-norbornanecarboxylic acid (BCH)	116.2 ± 6.2

Bacteria (10^9^ CFU/mL) were incubated for 1 h in the presence of 100 µM KYN containing 0.2 µCi ^3^H-KYN (see text for experimental details). Control values: 10.1 ± 1.2 fmoles. Data are the mean ± SEM of 3–4 experiments.

**Table 2:** Effects of selected endogenous and exogenous KAT inhibitors on the de novo synthesis of KYNA from L-KYN in live *L. reuteri*.

Putative Substrates and Inhibitors	KYNA Production (% of Control)
Glutamine (10 mM)	109.5 ± 1.2
α-Aminoadipate (10 mM)	110.9 ± 3.4
Aspartate (10 mM)	74.7 ± 3.7 **
AOAA (1 mM)	0.3 ± 0.6 ****
BFF-122 (1 mM)	30.3 ± 8.4 ****
PF-04859989 (100 µM)	8.8 ± 2.0 ****

Bacteria (10^8^ CFU/mL) were incubated for 3 h with 100 µM L-KYN and the respective test compounds (concentrations are indicated) as described in the text. Control values: 47.0 ± 2.7 fmoles/µL. Data are the mean ± SEM of 3–6 experiments. ** *p* < 0.01, **** *p* < 0.0001 vs. control values (one-way ANOVA, followed by Bonferroni’s post hoc test).

**Table 3:** Effects of various oxoacids (5 mM) on the neosynthesis of KYNA from L-KYN in live *L. reuteri*.

2-Oxoacids	KYNA Production (% of Control)
Pyruvate	131.8 ± 11.1
α-Ketoglutarate	123.7 ± 16.4
α-Ketoisocaproate	151.0 ± 9.6 **
α-Ketoisovalerate	188.7 ± 12.6 ****
Oxaloacetate	57.6 ± 4.9 *

Live *L. reuteri* (10^8^ CFU/mL) was incubated for 3 h with 100 µM L-KYN and the respective test compounds as described in the text. Control values: 51.2 ± 1.9 fmoles/µL. Data are the mean ± SEM of 4–6 experiments. * *p* < 0.05, ** *p* < 0.01, **** *p* < 0.0001 vs. control values (one-way ANOVA, followed by Bonferroni’s post hoc test).

**Table 4:** Effects of selected KAT substrates and inhibitors on KYNA synthesis from L-KYN in homogenized bacteria.

Putative Substrates and Inhibitors	KYNA Production (% of Control)
Glutamine (10 mM)	89.1 ± 3.7
α-Aminoadipate (10 mM)	79.6 ± 1.8 *
Aspartate (10 mM)	28.5 ± 2.2 ****
BFF-122 (1 mM)	5.9 ± 1.7 ****
PF-04859989 (1 mM)	12.2 ± 3.3 ****

Lysed *L. reuteri* (10^8^ CFU/mL) was incubated for 3 h with 100 µM L-KYN and the respective test compounds (concentrations are indicated). See text for experimental details. Control values: 112.1 ± 2.8 fmoles/µL. Data are the mean ± SEM of 3–9 experiments. * *p* < 0.05, **** *p* < 0.0001 vs. control values (one-way ANOVA, followed by Bonferroni’s post hoc test).

## Data Availability

Data contained within the article.

## Abbreviations

3-HK: 3-Hydroxykynurenine; ANA: Anthranilic acid; AOAA: Aminooxy acetic acid; KP: Kynurenine pathway; KYN: Kynurenine; KYNA: Kynurenic acid; LAT: Large neutral amino acid transporter; α7nACh: α7 Nicotinic acetylcholine; NMDA: N-methyl-D-aspartate
